# An AI-Based Image Quality Control Framework for Knee Radiographs

**DOI:** 10.1007/s10278-023-00853-6

**Published:** 2023-06-02

**Authors:** Hongbiao Sun, Wenwen Wang, Fujin He, Duanrui Wang, Xiaoqing Liu, Shaochun Xu, Baolian Zhao, Qingchu Li, Xiang Wang, Qinling Jiang, Rong Zhang, Shiyuan Liu, Yi Xiao

**Affiliations:** 1 Department of Radiology, Shanghai Changzheng Hospital, Naval Medical University, No.415 Fengyang Road, Huangpu District, Shanghai, 200003 China; 2 Deepwise Artificial Intelligence Laboratory, Beijing, 100089 China

**Keywords:** Artificial intelligence, Deep learning, Knee plain radiograph, Image quality control

## Abstract

Image quality control (QC) is crucial for the accurate diagnosis of knee diseases using radiographs. However, the manual QC process is subjective, labor intensive, and time-consuming. In this study, we aimed to develop an artificial intelligence (AI) model to automate the QC procedure typically performed by clinicians. We proposed an AI-based fully automatic QC model for knee radiographs using high-resolution net (HR-Net) to identify predefined key points in images. We then performed geometric calculations to transform the identified key points into three QC criteria, namely, anteroposterior (AP)/lateral (LAT) overlap ratios and LAT flexion angle. The proposed model was trained and validated using 2212 knee plain radiographs from 1208 patients and an additional 1572 knee radiographs from 753 patients collected from six external centers for further external validation. For the internal validation cohort, the proposed AI model and clinicians showed high intraclass consistency coefficients (ICCs) for AP/LAT fibular head overlap and LAT knee flexion angle of 0.952, 0.895, and 0.993, respectively. For the external validation cohort, the ICCs were also high, with values of 0.934, 0.856, and 0.991, respectively. There were no significant differences between the AI model and clinicians in any of the three QC criteria, and the AI model required significantly less measurement time than clinicians. The experimental results demonstrated that the AI model performed comparably to clinicians and required less time. Therefore, the proposed AI-based model has great potential as a convenient tool for clinical practice by automating the QC procedure for knee radiographs.

## Introduction

The knee joint is one of the largest and most complex joints in the human body and is subjected to strong gravitational forces [1]. Knee injuries can result from physical activities, aging, wear-and-tear, and various diseases, such as osteoarthritis (OA), rheumatic arthritis, spontaneous osteonecrosis of the knee (SONK), and knee instability [2–4]. Due to its frequency of injury, the knee joint is commonly examined in clinical practice. Anteroposterior (AP) and lateral (LAT) knee radiographs are currently the most commonly used imaging methods for assessing and diagnosing knee problems such as OA and SONK [4–8]. Kellgren and Lawrence [9] were pioneers in developing a classification system for osteoarthritis (OA) based on radiographs of the knee. They used AP knee radiographs and assigned a grade from 0 to 4 to each radiograph, with higher grades indicating increasing severity of OA. Subsequent research [10, 11] has shown that flexion radiographs (where the knee is flexed at 30 to 60°) can provide a more precise assessment of OA degeneration and narrowing, leading to more accurate diagnosis and treatment. Therefore, obtaining high-quality knee radiographs is crucial for the accurate diagnosis and treatment of knee diseases [6, 10, 11]. Clinicians’ decision-making regarding disease diagnosis and treatment can be compromised by low-quality radiographs, which can directly impact patient care [12]. However, the rejection rate for clinically qualified knee radiographs is often between 8 and 12%, indicating the need for improvements [13, 14].

Quality control (QC) is crucial in ensuring sufficient image quality for accurately diagnosing knee diseases. Typically, QC of knee joint radiographs involves quantitative measurements of imaging quality, such as the signal-to-noise ratio, level of sharpness, and number of artifacts, along with a number of positioning criteria. These criteria include the overlap ratio of the fibular head with the tibia on AP and LAT projections, the flexion angle on LAT projections, sufficient overlap of the femoral condyles on LAT projections, femoral and tibial condyles symmetry on AP projections, patella position on both AP and LAT projections, and visualization of the joint space. To qualify as clinically acceptable, knee joint radiographs must meet specific criteria [15], as follows:

- For AP knee radiographs, the following must be met: (1) The image should show the femoral and tibial condyles as well as the fibular head, with the articular surface in the center of the image. (2) The capitellum of the fibula should only slightly overlap with the tibia. (3) All bone textures of the knee joint should be clearly visible, and the surrounding soft tissue should be visible. (4) The knee joint should be fully displayed in the center of the image and parallel to the long axis of the image.
- For LAT knee radiographs, the following criteria must be met: (1) The knee joint space should be in the center of the image, and the femoral condyle and tibial plateau should overlap well. (2) The patella should be displayed laterally, with a clear gap with the femur, and the articular surface border should be sharp and without shadowing. (3) There should be minimal overlap of the femur and tibial plateau. (4) All bone textures of the knee joint should be clearly visible, as should the surrounding soft tissues.

Today, QC of knee joint AP and LAT radiographs is mainly performed through manual evaluation, which can be subjective and influenced by factors such as radiologist experience, cognitive level, fatigue, and environmental conditions, among others; thus, it can be challenging to meet clinical requirements with this approach [6, 10]. Therefore, there is an urgent need for automated, real-time radiograph quality analysis to assist technicians in determining the need for re-examination before the patient leaves the X-ray room, saving time and improving patient satisfaction [16].

Recent advancements in artificial intelligence (AI), particularly in deep-learning-based techniques, have enabled the development of convolutional neural networks (CNNs) with immense potential in various medical imaging applications such as recognition, classification, segmentation, diagnosis, and even decision-making [17–23]. With access to large amounts of labeled data, certain AI models based on deep learning have been shown to perform comparably or even better than human experts in assisting clinicians with disease screening and identification, resulting in improved work efficiency. Additionally, these models play a significant role in clinical education by enhancing the skills of junior radiologists [24, 25]. Previous studies have demonstrated the effectiveness of CNNs in performing image QC of chest radiographs [26–28], where the AI-based QC model automatically measured three quality criteria of AP chest radiographs: correct inclusion of lungs at all four edges, patient rotation, and inspiration. These studies found that the AI model achieved good agreement with clinicians, suggesting that the AI model can automate chest radiograph QC.

In this study, we aimed to investigate the feasibility of automated QC for knee joint radiographs using AI. We identified the three most critical and error-prone criteria for knee joint radiograph positioning, including the overlap ratio of the fibular head with the tibia on AP and LAT projections, as well as the flexion angle on LAT projections. The objective was to compare the performance of our proposed AI-based model with observations made by clinicians to assess whether the AI-based QC model can automate the output of clinicians in knee radiograph QC.

## Materials and Methods

### Ethics Statement

This study was approved by the Institutional Review Board of Shanghai Changzheng Hospital (2022SL071) before patient information was accessed, and the requirement for informed patient consent was waived due to the retrospective nature of the analysis and the anonymity of the data.

### Data Collection

We retrospectively collected 2,212 knee joint plain radiographs from 1208 patients from the Picture Archiving and Communication System (PACS) of Shanghai Changzheng Hospital (also referred to as Center 1) to train and validate the proposed AI model. Of these radiographs, 910 were AP radiographs, and 1302 were LAT radiographs. Specifically, 1638 plain radiographs from 796 patients (including 597 AP radiographs and 1041 LAT radiographs) were randomly selected as the training cohort, while the remaining 574 images from 412 patients (including 313 AP radiographs and 261 LAT radiographs) were used as the internal validation cohort. It is worth mentioning that we used a patient-wise partitioning strategy for the training and validation cohorts, ensuring that images from a single patient were only included in either the training or validation dataset, but not both.

**Fig. 1:**
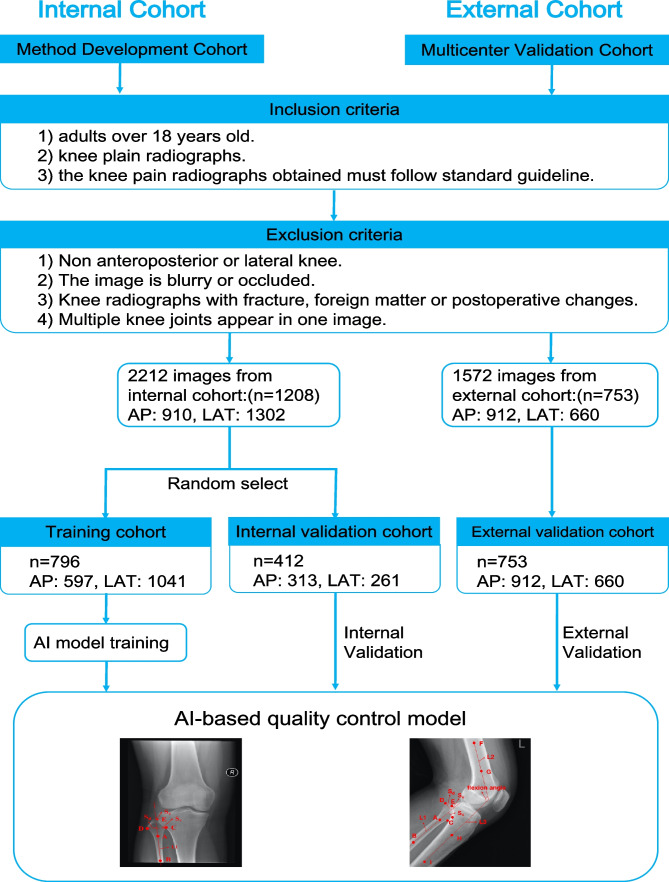
Inclusion and exclusion criteria for this study. A total of 3784 knee plain radiographs were used to train and validate the generalization performance of the proposed AI-based QC model

To further validate the generalizability of the proposed AI-based QC model across different hospitals, an independent external validation cohort was collected from six other hospitals (referred to as Centers 2–7) that included 1572 knee radiographs from 753 patients, including 912 AP radiographs and 660 LAT radiographs, as shown in Fig. 1. In this study, we focused on performing QC for individual images rather than patient disease diagnosis, and so QC performance was evaluated at the individual-image level rather than at the patient level.

The data collected for this study adhered to the following inclusion and exclusion criteria. Radiographs were included if they (1) were taken from patients over 18 years old; (2) were plain knee joint radiographs; and (3) were obtained in accordance with standard guidelines [29]. Radiographs were excluded if (1) they were not AP or LAT projections of the knee joint; (2) they were blurred or occluded, thus affecting the observation of knee joint structures; (3) the knee joint depicted on the radiograph exhibited fractures, foreign bodies, postoperative changes, or severe osteoarthritis; or (4) they showed multiple knee joints in a single image.

All images were captured using equipment from Philips, General Electric or Canon, and any sensitive information was fully anonymized. Table 1 shows the data distribution for all cohorts.

**Table 1:** Data distribution for different cohorts

	**No. of patients**	**No. of APs**	**No. of LATs**
**Training cohort** (Center 1)	796	597	1041
**Internal validation cohort** (Center 1)	412	313	261
**External validation cohort**	753	912	660
Center 2	20	20	16
Center 3	144	279	215
Center 4	168	174	111
Center 5	244	266	209
Center 6	83	81	51
Center 7	94	92	58

### Data Annotations

Plain knee radiographs are commonly used to diagnose knee joint diseases due to their ability to reveal the structural information of the knee. In this study, we selected three of the most critical and computationally challenging QC criteria for knee radiographs to evaluate the performance of an AI-based model against clinicians. These criteria are defined as follows:

1. Anteroposterior fibular head overlap ratio (AP overlap ratio): measures the overlap ratio between the fibular head and the tibia on AP knee plain radiographs.
2. Lateral fibular head overlap ratio (LAT overlap ratio): measures the overlap ratio between the fibular head and the tibia on LAT knee plain radiograph.
3. Flexion angle of the lateral knee (LAT flexion angle): measures the angle between the femur and the tibia on LAT knee plain radiograph.

To ensure the accuracy of the annotations, two associate chief musculoskeletal (MSK) radiologists with 10 and 13 years of experience first annotated all plain knee radiographs with key points. A committee of two chief MSK radiologists with 26 and 36 years of experience then reviewed all annotations and corrected any misplaced key points. Two other experts simultaneously reviewed all annotations, and any ambiguous labels were discarded. All annotations were then confirmed to be consistent and indisputable.

### Preprocessing

All AP/LAT knee radiographs were converted from raw DICOM format to npy format using Python and SimpleITK [30]. To enhance the visualization of skeletal features and remove redundant information, we adjusted the displayed details using window width and window level as calculated by adaptive histogram equalization with limited contrast.

### Computing of QC Criteria

Computing QC results for overlap ratios or flexion angle directly from images is challenging. To address this problem, we defined key points that describe the important positions of knee joints in an image. According to the QC requirements, for the AP knee plain radiographs, we used 5 key points, and for the LAT knee plain radiographs, we used 9 key points. Table 2 describes the definitions of these key points.

**Table 2:** Detailed description of key points

**AP/LAT**	**Key point**	**Description**
AP/LAT	A/B	Diaphyseal orientation of the fibula is determined by two points on the center of the fibula diaphysis: key point A in the mid-fibula and key point B in the distal fibula
	C	The key point on the head of the fibula closest to the tibia
	D	The key point on the fibular head furthest away from the tibia
	E	The key point where the fibular head overlaps the tibia
LAT	F/G	Diaphyseal orientation of the femur is determined by two points on the center of the femur diaphysis: key point F in the proximal femur and key point G in the mid-femur
	H/I	Diaphyseal orientation of the tibia is determined by two points on the center of the tibia diaphysis: key point H in the proximal tibia and key point I in the mid-tibia

**Fig. 2:**
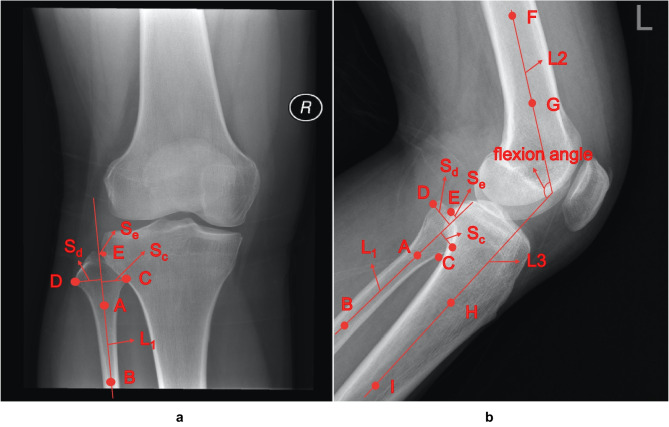
Example annotations of predefined key points and their corresponding auxiliary lines. **a** AP knee plain radiograph. **b** LAT knee plain radiograph. Auxiliary lines L1, L2, L3, vertical lines Sc, Sd, Se and flexion angle are all shown

Figure 2 shows examples of predefined key points (A–I) and their corresponding auxiliary lines on AP and LAT knee plain radiographs. The line connecting key points A and B represents the diaphyseal orientation of the fibula, defined as L_1_. The distance from key point C to line L_1_ is defined as Sc, the distance from key point D to line L_1_ is defined as Sd, and the distance from key point E to line L_1_ is defined as Se. The overlap ratio is calculated using $(S_{c}-S_{e})/(S_{c}-S_{d})$, as shown in Fig. 2a, if key points E and C are located on the same side of straight line L1; otherwise, it is calculated using $(S_{c}-S_{e})/(S_{c}-S_{d})$, as shown in Fig. 2b. The line connecting key points F and G represents the diaphyseal orientation of the femur, defined as L_2_. The line connecting key points H and I represents the diaphyseal orientation of the tibia, defined as L_3_. The LAT flexion angle is defined as the angle between line L_2_ and line L_3_.

It is important to note that key points A, B, F, G, I, and H are used to determine the diaphyseal orientation of the tibia, femur, and fibula. However, these key points are not unique, and slight movement along the diaphyseal orientation will not affect the finalization of the diaphyseal orientation. For instance, key points A and B can be slightly adjusted along line L_1_, but it is essential to ensure that the point is in the middle of the backbone cross-section (in the vertical direction of L_1_).

### The Proposed AI-Based QC Model

**Fig. 3:**
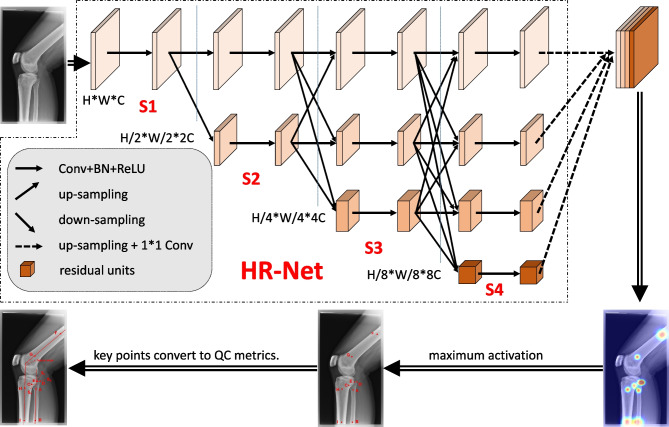
Pipeline of the proposed AI-based QC model

In this study, we used an HR-Net-based framework [31] to design our automatic QC model for knee joint radiographs, as shown in Fig. 3. Our model was trained to detect a set of predefined key points, and auxiliary lines were drawn to aid in the interpretation of key measurements, as precise values for knee flexion angle and overlap ratios are not directly available. Finally, we used a set of simple but effective geometric calculations to compute the overlap ratio of the fibular head with the tibia on AP and LAT projections, as well as the flexion angle on LAT projections.

More specifically, we first applied an HR-Net [31] model pretrained using ImageNet [32] as a feature extraction backbone to detect predefined key points (key points A–E for AP knee radiographs and A–I for LAT knee radiographs). Auxiliary lines were then drawn to interpret key measurements such as the diaphyseal orientation of the tibia, femur, and fibular head and the overlap between the fibular head and the tibia. Subsequently, geometric calculations were performed to calculate the overlap ratio of the fibular head and the tibia and the angle between the femur and the tibia.

As shown in Fig. 3, HR-Net is a parallel multiresolution and multibranch network framework that ensures semantic information interaction between different branches and maintains high resolution throughout the whole process. Here, semantic information refers to the computed image features at different scales. The model starts from a stem block that decreases the input resolution to 1/4 by using two stride-2 3 × 3 convolutions; the resulting image then serves as the input of the multiresolution and multibranch network. A high-resolution subnetwork is then used as the first stage (S1 in Fig. 3), and the previous high resolution is maintained (1/4 of the original input resolution) throughout the whole process. At each new stage, a high-to-low resolution stream is added in parallel and connected to the multiresolution streams. The later stages not only consist of the resolutions from the previous stage but also have an extra lower resolution stream. Four stages are applied in the whole process, and the number of channels C is doubled while the resolution gradually drops to half (i.e., C = 32, 64, 128, and 256 for feature maps F1, F2, F3, and F4, respectively).

To make better use of multiresolution information, an exchange model is used to exchange information across parallel subnetworks and is repeated several times (e.g., every 4 residual units; only 2 residual units are shown in Fig. 1). In the exchange model, information from different subnetworks is downsampled/upsampled to the same resolution, and 3 × 3 convolutions with stride 1 are used to maintain channel consistency. For example, if the feature $I_{r},r=1, 2, 3$ in stage 3 (S3) is associated with the output feature $O_{r},r=1, 2, 3$ after an exchange model, and the final output is the sum of the three inputs $o_{r}=\int^{r}_{1}(I_{1})+\int^{r}_{2}(I_{2})+\int^{r}_{3}(I_{3})$, where *r* is the resolution index, an extra output $o_{r}=\int^{4}_{1}(I_{1})+\int^{4}_{2}(I_{2})+\int^{4}_{3}(I_{3})$ is obtained across stages (from S3 to S4). The model repeats the information exchange across the multiresolution subnetworks, with S2, S3, and S4 containing 1, 4, and 3 exchange models, respectively. This enables more effective multiscale fusion learning and allows subnetworks with different resolutions to contribute different pieces semantic information, leading to a more expressive final feature map. Subsequently, features F2-F4 are converted to be consistent with feature F1 using upsampling and 1*1 Conv (H*W*C, F1 is only 1*1 Conv), and then features F1-F4 are concatenated as O1. Finally, a 1 * 1 conv is used to obtain the final output with shape H*W*9. Afterward, the location with the highest probability (maximum activation) in the output probability map is considered the detected key point.

### Implementation Details

In this study, we used the mean square error (MSE) loss to measure the deviation between the regressed heatmaps and the ground-truth heatmaps, which were generated using a 2D Gaussian distribution with sigma = 2. It should be recalled that the LAT knee joint radiograph has four additional key points over the AP knee joint. To manage this difference, we set the regression objective to 0 for these four key points on the AP knee radiographs. This approach offers two benefits: the model can handle both AP/LAT knee radiographs, and the input image can be automatically identified as an AP or LAT knee radiograph based on the number of detected key points.

We trained the model using stochastic gradient descent (SGD) with an initial learning rate of 0.002, which decayed by 10 after 50 epochs and 56 epochs. The momentum was set to 0.9, and the weight decay was set to 0.0001. We used a mini-batch size of 4 and trained the model for a total of 60 epochs. The short side of the input image was resized to 288 while keeping the original aspect ratio. To increase the diversity of the data, data augmentation strategies including random flips and random inversions with a probability of 0.5 were used. Experiments were implemented using the open-source toolbox mmdetection and pytorch [33]. To speed up training, we used four NVIDIA 1080TI GPUs to train our model.

## Results

### Primary Validation

We evaluated the performance of the proposed AI-based QC model by measuring its agreement with clinicians using the intraclass correlation coefficient (ICC) [34]. We chose two-way random effects, absolute agreement, and a single rater as our measurement model, abbreviated as ICC(2,1) [34]. ICC > 0.75 indicates good reliability, and ICC > 0.9 indicates excellent reliability. *p* values less than 0.05 were considered to indicate statistical significance using independent-samples t tests.

As shown in Table 3, the ICCs of the proposed AI-based QC model and clinicians in the internal validation cohort were 0.952 (95% confidence intervals (CI): 0.94–0.96), 0.895 (95% CI: 0.87–0.91), and 0.993 (95% CI: 0.99–0.99) for the AP overlap ratio, LAT overlap ratio, and LAT flexion angle, respectively. There were no statistically significant differences between clinicians and the AI-based model on any of the three criteria, namely, AP overlap ratio (*p* = 0.498), LAT overlap ratio (*p* = 0.858), and LAT flexion angle (*p* = 0.777). For the external validation cohort, the mean ICCs between clinicians and the AI-based model were 0.934 (95% CI: 0.92–0.94), 0.856 (95% CI: 0.83–0.88), and 0.991 (95% CI: 0.99–0.99) for the AP overlap ratio, LAT overlap ratio, and LAT flexion angle, respectively. Similarly, there were no statistically significant differences between clinicians and the AI-based model in terms of AP overlap ratio (*p* = 0.093), LAT overlap ratio (*p* = 0.278), and LAT flexion angle (*p* = 0.632). These results demonstrate that the QC performance of the proposed AI model is comparable to that of clinicians when testing on data within and across different centers, indicating great potential for application in clinical practice.

**Table 3:** ICC measurements between clinicians and the AI-based model in terms of AP overlap ratio, LAT overlap ratio, and LAT flexion angle

**Data Sources**	**AP overlap ratio (95% CI)**	**LAT overlap ratio (95% CI)**	**LAT flexion angle (95% CI)**
**Internal validation cohort**			
Center 1	0.952 (0.94–0.96)	0.895 (0.87–0.91)	0.993 (0.99–0.99)
**External validation cohort**			
Center 2	0.913 (0.79–0.96)	0.909 (0.66–0.97)	0.984 (0.87–1.0)
Center 3	0.915 (0.86–0.94)	0.874 (0.83–0.91)	0.976 (0.97–0.98)
Center 4	0.930 (0.91–0.95)	0.869 (0.81–0.91)	0.978 (0.91–0.99)
Center 5	0.940 (0.92–0.95)	0.827 (0.78–0.87)	0.997 (0.99–1.0)
Center 6	0.934 (0.90–0.96)	0.877 (0.80–0.93)	0.993 (0.99–1.0)
Center 7	0.911 (0.87–0.94)	0.825 (0.72–0.89)	0.983 (0.95–0.99)
Mean	0.934 (0.92–0.94)	0.856 (0.83–0.88)	0.991 (0.99–0.99)

**Fig. 4:**
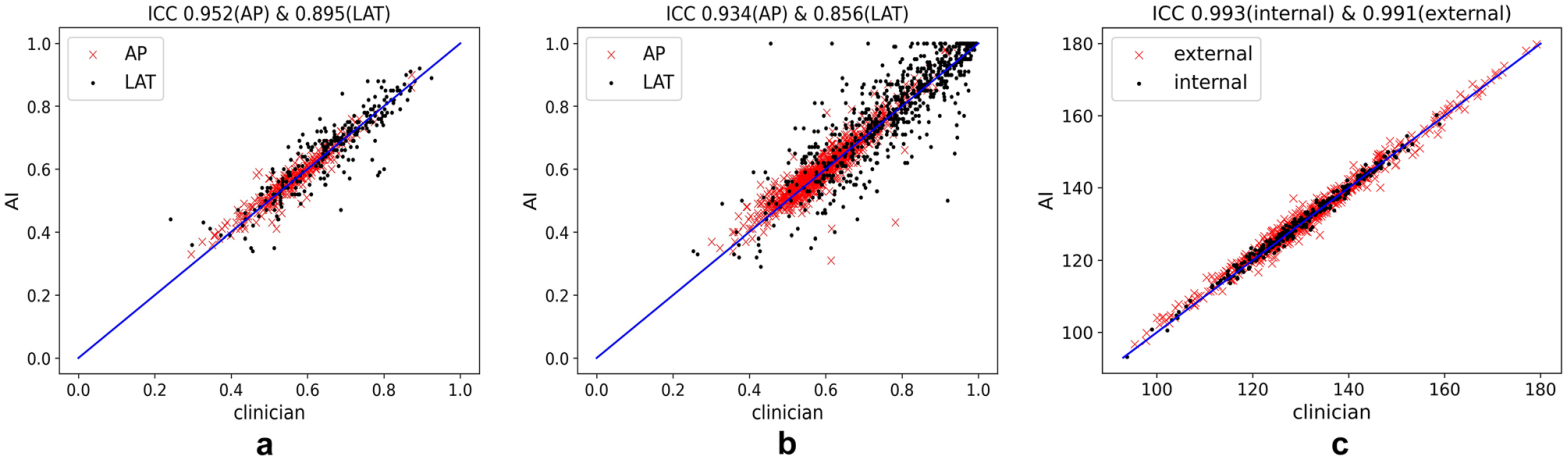
Scatter plots of the correlations between the AI model and clinicians. **a** AP/LAT overlap ratios in the internal validation cohort, **b** AP/LAT overlap ratios in the external validation cohort, **c** LAT flexion angles in both the internal and external validation cohorts

Figure 4 illustrates the correlation between the AI-based model and clinicians in terms of AP/LAT overlap ratios and LAT flexion angle. Specifically, Fig. 4a, b depicts the scatter points for the AP/LAT overlap ratios in the internal and external validation cohorts, respectively, while Fig. 4c shows the scatter points for the LAT flexion angle in both cohorts. The blue line in the center of each plot indicates exact agreement between the AI model and clinicians, meaning no deviation between the two.

In general, the scatter points for the AP/LAT overlap ratios in Fig. 4a, b were closer to the centerline on the AP knee radiographs, suggesting a slight deviation. However, on the LAT knee radiographs, the scatter points were more spread out relative to the centerline. This pattern was consistent across both internal and external validation cohorts. On the other hand, Fig. 4c shows high agreement between clinicians and the AI-based model for LAT flexion angle, with little deviation in either cohort.

To further quantify the agreement between the AI-based model and clinicians, Table 4 presents the mean, standard deviation, and maximum deviation of the AP/LAT overlap ratios in the internal and external validation cohorts. Notably, the mean, standard deviation, and maximum deviation of the LAT overlap ratio were consistently larger than those of the AP overlap ratio in both cohorts, which aligns with the scatter plots in Fig. 4. Since the LAT flexion angle ranges from 0 to 180, normalized values were also included in Table 4 for a fair comparison. The normalized values demonstrate significant agreement between clinicians and the AI-based model in terms of LAT flexion angle, with more agreement observed in the internal validation cohort, which is also consistent with the findings shown in Fig. 4.

**Table 4:** Means and standard deviations of absolute deviations between clinicians and the AI-based model in both the internal and external validation cohorts

	AP overlap ratio	LAT overlap ratio	LAT flexion angle*
**Internal validation cohort**			
Mean	0.019	0.040	1.049(0.006)
Standard deviation	0.018	0.039	0.748(0.004)
Max deviation	0.117	0.217	3.730(0.021)
**External validation cohort**			
Mean	0.024	0.058	1.289(0.007)
Standard deviation	0.026	0.058	1.109(0.006)
Max deviation	0.352	0.544	8.582(0.477)
*p* value	< 0.01	< 0.01	< 0.01

*The AP/LAT overlap ratio ranges between 0 and 1, while the LAT flexion angle ranges between 0 and 180; the numbers in parentheses represent normalized angles, ranging between 0 and 1. The *p* value was calculated based on the mean using t tests

### Comprehensive Performance Analysis

**Fig. 5:**
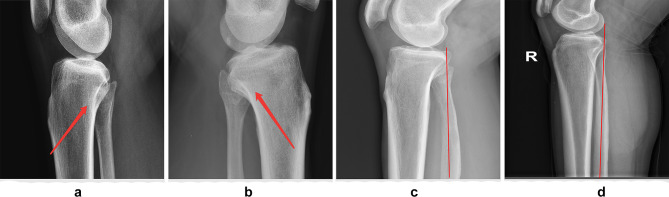
Visualization of occlusion and variation of the fibular head. **a** and **b** show occlusion of the fibular head, making the location of key point C ambiguous and resulting in inaccurate LAT overlap ratios. In **c** and **d**, the fibular head is bent due to variations, where the red line indicates the orientation of the fibula. The curved fibular head distorts the calculations of Sc, Sd, and Se, resulting in inaccurate LAT overlap ratios. Due to occlusion and variation, the fibular head overlap ratio is generally less consistent on LAT knee radiographs than on AP knee radiographs

Our primary validation results showed that the proposed AI-based QC model performed poorer on LAT radiographs than on AP radiographs. Through a comprehensive visual analysis of knee plain radiographs, we found that occlusions were relatively common on the LAT knee joint radiographs, as shown in Fig. 5a, b, making identifying key point C difficult and resulting in inaccurate LAT overlap ratios. Additionally, the fibular head is prone to variations, such as the distortions shown in Fig. 5c, d, resulting in deviations in the final measurement results. Due to occlusion and variation, the fibular head overlap ratio is generally less consistent on LAT knee radiographs than on AP knee radiographs. In summary, the deviations on AP knee radiographs are generally lower than those on LAT knee radiographs, mainly due to the relatively better clarity and visibility of the knees on the AP projections.

### Performance in Key Point Detection

Since HR-Net-based key point detection is the basis of the proposed AI-based QC model, we reported the quantitative performance of the key point detection model in terms of average precision (AP) and average recall (AR) [35]. Object key point similarity (OKS) was used to measure the deviation in the key points, calculated as $OKS=\frac{\sum_{i}\text{exp}(-d^{2}_{i}/2s^{2}k^{2}_{i}\delta(v_{i}>0))}{\sum_{i}\delta(v_{i}>0)}$. Here, *s2* is the object scale, which we set as the area of the smallest bounding box containing all key points; *di* is the Euclidean distance between a detected key point and its corresponding ground truth; *vi* is the visibility flag; and *ki* is a predefined constant derived from the statistics of annotation deviations. Generally, ${\mathrm{k}}_{\mathrm{i}}=2{\upsigma }_{\mathrm{i}}$, where ${\upsigma }_{\mathrm{i}}$ is the standard deviation, which differs for different key points. We applied the mean of the statistical results of the key point detection statistics [35]; that is, ${\upsigma }_{\mathrm{i}}$ for key points A–I were [0.083, 0.083, 0.029, 0.029, 0.029, 0.083, 0.083, 0.083, 0.083].

**Fig. 6:**
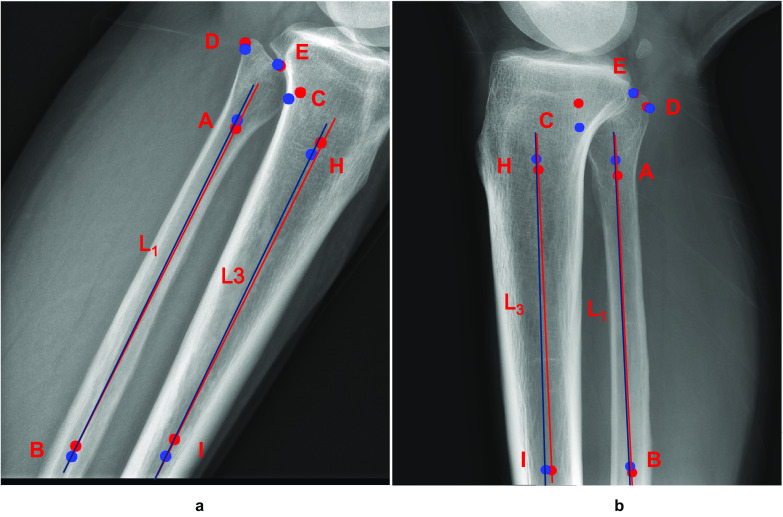
Visualization of key point detection, where the red key points and lines are from the clinician’s annotations and the blue key points and lines were generated by the AI model. Both occlusion and blurring affected the identification of key point C

As shown in Table 5, our experimental results showed that our key point detection model achieved excellent mean average precision (mAP) values. Figure 6 also visualizes examples of key point detection results, where red key points and lines are clinician annotations, and blue key points and lines represent the AI model’s generated results. As expected, we observed that occlusion and ambiguity affected the identification of key point C, which could lead to inaccurate measurement results.

**Table 5:** Performance in key point detection

		mAP*	AP50	AP75	mAR*	*p* value
AP	Internal validation cohort	0.988	0.989	0.989	0.996	< 0.01
	External validation cohort	0.922	0.986	0.986	0.955	
LAT	Internal validation cohort	0.846	0.990	0.972	0.903	< 0.01
	External validation cohort	0.788	0.990	0.922	0.852	

*mAP is the mean average precision, mAR is the mean average recall, and the *p* value is calculated based on the mAP using *t* tests

## Discussion

In this study, we proposed an AI-based fully automatic QC model for knee radiographs. The model uses HR-Net to identify predefined key points in images and then performs a set of geometric calculations to transform these key points into three QC criteria: the AP overlap ratio, LAT overlap ratio, and LAT flexion angle. The proposed model was trained and validated using a total of 2212 knee plain radiographs, including 910 AP radiographs and 1,302 LAT radiographs. An additional 1572 knee radiographs, including 912 AP radiographs and 660 LAT radiographs, were also collected from six external centers as an external validation cohort.

Our results demonstrated that the proposed AI-based model achieved similar reliability to that of clinicians on all three QC criteria. In the internal validation cohort, the ICCs for the overlap ratios of the AP fibular head and LAT fibular head and the LAT flexion angle were 0.952, 0.895, and 0.993, respectively, while the corresponding ICCs for the external validation cohort were 0.934, 0.856, and 0.991. Our experimental results demonstrated that the differences in the performances between clinicians and the AI-based model on all three QC criteria in the internal and external validation cohorts were not significant. The proposed model was substantially more efficient, taking an average of 0.52 $\pm$ 0.10 (AP)/0.52 $\pm$ 0.10 (LAT) seconds to process a knee plain radiograph versus the 15.23 $\pm$ 1.33 (AP)/24.49 $\pm$ 1.91 (LAT) seconds required by clinicians. Therefore, the proposed AI-based QC model has great potential as an effective and efficient auxiliary tool to help clinicians reduce the time and effort in performing QC while maintaining objective, consistency, and comparable accuracy.

However, this study also had several limitations. First, we found statistically significant differences between the internal and external validation cohorts on all three QC criteria and our key point detection in our experimental results. This outcome was expected, given that the training and internal validation cohorts were obtained from the same center, whereas the external validation cohort was sourced from six other centers. Increasing the diversity of data sources in the training cohort could address this issue. Second, the performance in key point detection needs to be improved, especially on LAT knees, due to factors such as occlusion, blur, and variation that can affect the detection of key points. Additionally, key points A, B, F, G, H, and I, located along the diaphyseal orientation, are not well defined, and annotations may vary between clinicians. Further exploration of other methods of determining the diaphyseal orientation is necessary. As a feasibility study, we only investigated three QC criteria for image positioning, and more quantitative measures, including imaging quality and other positioning criteria, will be explored in the future for a more complete, clinically applicable QC system. Finally, other quantitative metrics will also be explored to measure agreement between clinicians and the AI-based model.

In conclusion, the proposed AI-based QC model, by incorporating three objective QC criteria, including the AP overlap ratio, LAT overlap ratio, and LAT flexion angle, achieved reliability comparable to that of clinicians. In clinical practice, clinicians are often too busy to carefully measure knee radiographs. The proposed AI-based QC model can automate the QC of knee radiographs with a performance that is highly consistent with traditional manual evaluation but more efficient. Therefore, the proposed AI-based model has great potential for automating the QC of knee radiographs by clinicians while offering great conveniences to clinical practice.

## Data Availability

The data that support the findings of this study are available on request from the corresponding author. The data are not publicly available due to privacy or ethical restrictions.

## Abbreviations

AI: Artificial Intelligence
AP: Anteroposterior
LAT: Lateral
ICCs: Intraclass consistency coefficients
CNNs: Convolutional neural networks
QC: Quality control
CI: Confidence intervals
SONK: Spontaneous osteonecrosis of the knee
